# Supporting Pacific Island Countries to Strengthen Their Resistance to Tobacco Industry Interference in Tobacco Control: A Case Study of Papua New Guinea and Solomon Islands

**DOI:** 10.3390/ijerph10083424

**Published:** 2013-08-06

**Authors:** Judith McCool, Jeanie McKenzie, Annabel Lyman, Matthew Allen

**Affiliations:** 1 School of Population Health, Faculty of Medical and Health Science, University of Auckland, 22 Princes Street, Auckland 1010, New Zealand; 2 Secretariat of the Pacific Community, BP D5 98848 Noumea, New Caledonia; E-Mail: JeanieM@spc.int; 3 Framework Convention Alliance, Palau; E-Mail: lymana@fctc.org; 4 Allen and Clarke Policy and Regulatory Specialists Ltd., PO Box 10730, Wellington 6143, New Zealand; E-Mail: mallen@allenandclarke.co.nz

**Keywords:** tobacco industry, FCTC Article 5.3, Pacific Islands, policy

## Abstract

Tobacco use is the biggest single preventable cause of non-communicable diseases (NCDs) in the Western Pacific region. Currently, 14 Pacific Island countries have ratified the WHO Framework Convention on Tobacco Control (FCTC) and, in having done so, are committed to implementing tobacco control measures aligned with the FCTC. Progressing strong and effective tobacco control legislation is essential to achieving long term gains in public health in small island countries. However, survey evidence suggests that pervasive tobacco industry interference serves to undermine tobacco control and public policy in several Pacific countries. An initiative was developed to provide dedicated, in-country technical support for developing legislation and policy to support implementation of Article 5.3 of the FCTC in the Solomon Islands and Papua New Guinea. This paper examines the factors that have assisted the two Pacific countries to make progress in implementing Article 5.3 and what this might mean for supporting progress in other Pacific settings. A document analysis was undertaken to identify the process and outcome of the intervention. Two significant outputs from the project including having identified and documented specific examples of TII and the development of draft legislation for Article 5.3 and other key resources for public servants both within and outside the health sector. Key determinants of progress included a motivated and engaged Ministry of Health, active civil society group or champion and access to media to prepare tobacco industry related material to stimulate public and policy sector debate.

## 1. Introduction

Tobacco use is the biggest single preventable cause of non-communicable diseases (NCDs) in the Western Pacific region. The WHO Framework Convention on Tobacco Control (FCTC) serves as the most influential global treaty on reducing the demand for, and supply of, tobacco products. Within the Western Pacific region, 14 Pacific Island countries (PICs) have ratified the treaty and in having done so have committed to implementing tobacco control measures aligned with the FCTC and objectives. There has been considerable progress in legislation and policy in the tobacco control area, but challenges in relation to adequate enforcement still remain. Reasons for the impeded progress of the FCTC vary by country; some argue it is due to lack of funding and technical support to advance legislation [1,2,3], Martin also identified resourcing/staff capacity, particularly for enforcement; limited whole-of-government networking and commitment (*i.e.*, beyond the health sector); and limited anti-tobacco advocacy from NGOs as key challenges [4]. Tobacco industry inference (TII) has received little attention in its role as in applying resistance to tobacco control in the Pacific Islands [5].

British American Tobacco (BAT) and Philip Morris (PM), two of the largest tobacco trans-nationals active in the Pacific, are facing increasing challenges to tobacco sales and marketing due to tightening tobacco control regulations brought about through the FCTC. However, many countries still provide a relatively open environment for the tobacco industry to grow their market. The latest BAT interim report reveals the challenges they face in the current economic environment. However, in spite of this they report to have “grown revenue, our pricing momentum remains strong and our Global Drive Brands continue to perform well” [6]. This globalized marketing strategy lies at the heart of their global campaign to invest in developing and emerging economies [7]. Due to their relatively small and geographically isolated position, the Pacific Islands are vulnerable to tobacco industry interference in policy making [8]. Tobacco industry strategies invariably include; attempts to influence people in power, providing advice to undermine effective legislation and policy, attempts to discredit reputable research [7,9,10], investing in corporate social responsibility activities and attempting to undermine bans on advertising, promotion of products (e.g., cross-border advertising). These strategies, often legitimized in the interests of promoting global trade and economic development, have flourished across the Pacific region, bringing greater urgency to secure legislation that protects populations from the influence of the tobacco industry [10,11,12].

The FCTC specifically requires that, “in setting and implementing their public health policies with respect to tobacco control, Parties shall act to protect these policies from commercial and other vested interests of the tobacco industry in accordance with national law” [11]. Given both anecdotal and the formative survey on TII in several Pacific Island countries, the aim of this initiative was to examine the impact of a tailored technical assistance approach to strengthen countries response to interference in policy making. This paper examines the factors that have assisted two Pacific countries to make progress in implementing Article 5.3 and what this might mean for supporting progress in other Pacific settings. 

## 2. Experimental Section

### 2.1. Aims and Objectives

The aim of this paper is to (a) map the process undertaken to develop Pacific-relevant technical support for resisting tobacco industry interference and (b) document the key elements instrumental in strengthening the Solomon Islands’ and Papua New Guinea’s policy response to tobacco industry interference. 

### 2.2. Method

A document analysis of all text based resources and correspondence gathered prior to and throughout the intervention with both participating countries. This process was undertaken to establish an appreciation of the key and associated factors that contributed to the objectives of this initiative. This was achieved by firstly: (a) by assessing knowledge, awareness and compliance with FCTC Article 5.3 in 14 PICs (described elsewhere), then (b) reviewing in-country consultation and assessment of legislation and policy status and nature of industry activity in order to (c) identifying changes to administrative procedures, policies and legislation and putting into practice government level policy, ‘code(s) of conduct’ and/or appropriate legislation.

The process for reviewing the data (document, emails, records of events and other textual details from the intervention) were retrieved from electronic archives and systematically reviewed. This data was collected by the team and other stakeholders and therefore was collated by the research assistant (TP) in the process of the analysis.

### 2.3. Success Case Method (for Evaluation of Process and Outcomes of Intervention)

For this project, the methodology that was selected as most appropriate was the Success Case Method (SCM) [12]. This project, which aims at supporting Pacific Island countries to strengthen their resistance to the tobacco industry, used the SCM to portray the elements of the intervention that were considered to be essential or instrumental to the ‘success’ of that program or initiative. Through the use of the SCM, the characteristics and events that took place are captured to identify what worked well from the initiatives of this project and ultimately, what, under alternative circumstances and conditions, could be improved. 

The SCM is appropriate for evaluating this project because it provides a cost effective and reliable way of highlighting the process that successfully achieved the pre-stated objective to support Pacific Islands to gain awareness of and resources to effectively counteract TII in public health policy. This methodology also identifies the components that enabled or challenged the countries’ capacity to successfully adopt the initiatives of this project, ultimately making it a success case.

To appraise the process and impact of the intervention, we systematically reviewed emails, newspaper articles, minutes, interim reports, resources prepared for government departments on FCTC Article 5.3, implementation plans, proposals, drafts, legislation and background reports. Electronic versions of all documents were made available by the project team for the research assistant to collate for the preliminary review. Together these documents recorded the events that took place in-country (Solomon Islands and Papua New Guinea), the correspondence between the project team and the country governments, and the team-work between the members of the project team. The interim reports, regularly provided to the funder, were especially helpful in documenting the progress of the project and tracking the project objectives that were being fulfilled. By carefully reviewing these documents, evidence of the process of development of liaison with countries, development of the intervention (the ‘toolkit’) and the in-country meetings and drafting of legislation) was extrapolated. 

### 2.4. Objectives of Intervention

The following set of objectives were established and agreed as an appropriate series of steps for progressing implementation of FCTC Article 5.3: 

OBJECTIVE 1: To conduct preliminary discussions with three Pacific Island countries and establish a shared vision. 

OBJECTIVE 2: To identify key partners, develop advocacy plans and instigate the first round of in-country meetings and consultations. 

OBJECTIVE 3: To develop a draft toolkit tailored for the Pacific Island setting that will include guidance for addressing tobacco industry interference. 

OBJECTIVE 4: To develop and finalize model legislation for protection against tobacco industry interference in public health.

OBJECTIVE 5: To provide on-going technical advice and support to assist PICs in responding to tobacco industry interference. 

OBJECTIVE 6: To ensure documentation and dissemination of key outputs and examples of good practice, to encourage adoption of tools and good practices. 

### 2.5. Countries Selected

Fourteen Pacific Parties to the FCTC were asked to indicate their interest in receiving tailored support for managing tobacco industry interference by way of an on-line survey. Two countries opted to proceed to the intervention phase. The countries selected and invited to participate in the intervention were Papua New Guinea and Solomon Islands. Vanuatu was proposed as a third country but timing and resources precluded their participation in this round. Selection was also based upon previous direct requests for assistance from Vanuatu and Solomon Islands to address TII activities. 

### 2.6. The Process (Activities Undertaken)

The intervention was based around the concept of building partnerships as a platform for developing a tailored set of communication tools such as presentation/education materials to assist countries to effectively manage tobacco industry interference. The two countries were willing to participate in a project that sought openness about the mechanisms by which the industry is operating in public policy and the current level of interactions with the industry, with the view to working collaboratively to develop practical solutions for the future. A detailed profile of the activities undertaken in each setting is presented in Table 1. 

Preliminary discussions were conducted in the Solomon Islands between the Ministry of Health and the Tobacco Task Force and the project team, allowing initial action plans to be developed. To support these discussions, key partners from the Solomon Islands were identified. These key partners and the stakeholders were involved in country consultations and meetings at allied events such as WPRO or tobacco control meetings (opportunism). In alignment with Objective 3, several resources were developed that assisted in educating key partners about the nature and type of industry interference likely to be experienced in the Pacific and how to respond appropriately in accordance with FCTC Article 5.3. These resources included presentations about: the tobacco industry, understanding corporate social responsibility (CSR), the WHO Framework Convention on Tobacco Control (FCTC Article 5.3) [11] and countering tobacco industry interference. Guidelines, factsheets and legislation drafts were also developed as useful resources for participating countries. The specific activities, lead partners and outcomes from the intervention are presented in Table 1. 

**Table 1:** Project team, activities and outcomes.

Team	
Bloomberg Project Team	Jeanie McKenzie—Secretariat of the Pacific Community Annabel Lyman—Framework Convention Alliance Matthew Allen—Allen and Clarke Policy and Regulatory Specialists Judith McCool—Global Health, University of Auckland.
Collaborating Organisations Solomon Islands	World Health Organization Ministry of Health Solomon Islands Treasury Tobacco Control Task Force
Collaborating Organisations PNG	World Health Organization Ministry of Health Department of Personnel Management University of Papua New Guinea
Intervention in country	
“Contents of toolkit”	WHO Framework Convention on Tobacco Control (FCTC Article 5.3), Information on countering tobacco industry interference guidelines was included in the form of factsheet Draft FCTC Article 5.3 legislation [13]. PowerPoint presentation on TII created by team
Visit to Solomon Islands and activities	Visits Pre-visit engagement Two in-country visits (June 2012; August 2013) Follow-up correspondence (via email) Activities Meetings with key staff from WHO, MoH, Department of Public Service, Treasury staff and Civil Society (Tobacco Control Taskforce). Initial draft of legislation for Article 5.3 Reviewed Public Service Code of Conduct Assisted with drafting media response to TI article in Solomon Star newspaper.
Outcomes of visit to Solomon Islands	Completed ‘Article 5.3 Guidelines Compliance Assessment’ template, with Solomon Islands Tobacco Control Taskforce Developed and provided PowerPoint presentations on TII for Solomon Islands Public health team to utilize during ongoing awareness-raising visits with government departments Reviewed Tobacco Act and Regulations Drafted proposed Amendment to Tobacco Act on Tobacco Industry Interference Reviewed Code of Conduct for Ministry of Public Service Developed draft Government Policy Directive on Preventing Tobacco Industry Interference Developed draft Guidelines on Engagement with the Tobacco Industry Developed fact sheet on tobacco industry interference and importance of confronting it Publication of press release in response to TI statements on tobacco control bill in Solomon Star on World No Tobacco Day 31 May.
Number and timing of visit to PNG	Visits: Pre-visit engagement and consultation via Skype and email Three in-country visits July 2012, October 2012, June 2013 Post-visit correspondence (via email) Activities: In-country partners, including government, non-government and corporate sectors identified and invited to attend stakeholders awareness arising meeting (during second visit) Development of resources including Factsheets and short video [14] for use in public servant and civil society TII awareness raising workshop. Meeting to raise awareness on WHO FCTC Article 5.3 held (additional meeting with greater civil society representation held in October 2012) Ongoing activity in area of TII identified and meetings held to address this (e.g., meeting with Dean at UPNG to discuss tobacco industry educational sponsorships) Department Personnel Management initial discussion regarding redrafting of Code of Conduct and process for increasing awareness of Article 5.3 issues within public service Consultation meeting to amend draft legislation on Article 5.3 (and other relevant tobacco control provisions)
Outcome of visit to PNG (from visit one and two)	Assessment of level of tobacco industry interference across range of government departments undertaken (Treasury, Health, Customs) and review of existing MOU’s, codes of conduct. Amended draft legislation on Article 5.3 (and other relevant tobacco control provisions) Drafted factsheet on Article 5.3 for government and civil servants Drafted outline of monitoring activities for Article 5.3 Established plan of action for second visit to include NGO meeting, development of General Orders/Code of Conduct, Process Guidelines and progressing of legislation Presented key documents developed since first meeting to Department of Personnel Management including; factsheet on Article 5.3 for government and civil servants, Draft General Orders and Draft Guidelines for TI Interactions Established process and timeline for passage of legislation

## 3. Results and Discussion

### The Initiative Impacts

#### Solomon Islands

Four members of the project team visited the Solomon Islands for the first trip between 29 May and 1 June 2012. During this initial visit, several meetings were conducted with Ministry of Health members; Director of Public Health, Undersecretary for Health and the Public Health team. A meeting with the Tobacco Control Taskforce and the Ministry of Public Service was also conducted. Meeting with the Tobacco Control Taskforce allowed the project team to introduce the project and then formalize an agreement on the process for the project. In terms of technical assistance, the team was able to assist in: completing an ‘Article 5.3 Guidelines Compliance Assessment’ template, developing a PowerPoint presentation for the Public Health team to use when meeting with government departments, reviewing the Tobacco Act and proposed Regulations and the draft Code of Conduct for the Ministry of Public Service. Relevant documentation for the project was also collected or requested. 

From the reviewed documents and discussions with members of the Project team, it was evident that good timing and opportunism are ideal partners when advancing tobacco control policy. For example, on the day of the formative meeting of the staff and the project team, the Solomon Islands Tobacco Company (SITCO) had visited the Ministry of Health key policy makers to oppose the progress of the pending tobacco control bill (due to be passed that week). The project team was then able to provide the technical support, advocacy and policy advice that the Solomon Islands needed in order to address this interference. Furthermore, the team was in the Solomon Islands on World No Tobacco Day (WNTD) (31 May) so team members were able to participate in the ‘Say No to Tobacco Industry Interference’ initiatives. One of the unanticipated outcomes was that the team was able to develop and publish a press release in response to a Tobacco Industry article in the newspaper, which threatened against enforcing the Tobacco Act, as planned [15]. With the timing of this trip coinciding with WNTD related events; the Solomon Islands government was receptive of the initiatives the project provided. The initial impact of this interaction was significant in raising consciousness among the policy makers and the wider public (when it resulted in a news media exchange). Factors including the timing of events, government structures, Solomon Islands political environment, the availability of project team members and the responsiveness of the Solomon Islands key personnel were instrumental in the initial phase. 

Following the initial trip and with all the information gathered, several recommendations were then formed. These recommendations focused on reviewing and providing templates and guidelines to combat tobacco industry interference, and raising awareness and educating the public sector on tobacco industry interference. 

During the period of the second interim report to the funder, project objectives had either been fulfilled, or anticipated to be met in early 2013. Preliminary discussions had been made and key partners had been identified. However, several partnerships were currently in the process of being built. Other resources were developed during this reporting period, such as a factsheet informing public servants about Article 5.3. A few key documents were still in preparation: revision of the public service Code of Conduct, a policy paper for the National Executive Council and additional documents on training civil servants on Article 5.3. It is anticipated that the production of these supporting documents will contribute to the development of a resource toolkit that other countries can use. 

#### Papua New Guinea

Papua New Guinea presented several opportunities for strengthening legislation around TII. Specifically the opportunity afforded by a new Tobacco Act being drafted, allowed the project team to assess and contribute to the proposed inclusion of provisions relating to Article 5.3 of the FCTC. Secondly, the current status of TII in PNG was demonstrated by the revelation of several standing agreements, including one relating to tobacco tax restrictions between the Department of Finance and a tobacco company, another describing a scholarship funded by BAT to support University of Papua New Guinea students, and a third an agreement to allow the sale of “kiddi packs” (packs of 10 cigarettes). 

Consultation meetings both with PNG Government departments and NGOs were established prior to the visit to PNG to establish the current status of awareness about TII, strengthening existing Code(s) of Conduct among tobacco control, health, public service sectors and NGOs (civil society). The purpose of the intervention was to provide dedicated support to advance (either by talking through the details or drafting) legislation and policy instruments, the culmination of opportunities for exposing TII and using these events for demonstrating alternative responses that align with FCTC Article 5.3.

## 4. Discussion

Article 5.3 is a pivotal element of the FCTC as it directly implicates government and civil society in terms of their connection with tobacco industry activities and their capacity to action all other articles in the treaty. The review of the documents relating to the initiation and in-country engagement with the Solomon Islands and Papua New Guinea government identified several unscripted but serendipitous impacts. Specifically, in respect to Solomon Islands, the timing of the trip meant that the team was in country when the SITCO staff paid an unexpected visit to the Ministry of Health to discuss the introduction of the revised Tobacco Act. Secondly, the team was able to participate in WNTD. These two events coincided to spark a series of articles and debate in the national newspaper, with the tobacco company claiming that the introduction of the Tobacco Act would undermine economic development and employment in the Solomon Islands. The Ministry of Health responded with the following comment: to “bring about the No Tobacco legislation and implementing its enforcement is a very responsible thing to do” [16]. The timing of the SITCO visit to the Ministry of Health was aligned with the pending passing of the Tobacco Control Bill (which was due to be passed in conjunction with the WNTD celebrations). This turn of events illustrated the need for explicit guidelines about government engagement with the tobacco industry. Furthermore, these guidelines are needed to be driven, at the outset, by the health sector, including civil society but then broadened to other public service sectors. Gaining the support of the Chamber of Commerce and trade sectors is likely to meet with greater resistance. Prudent timing, seizing media exposure of tobacco industry activities, rallying public media and maintaining close engagement with civil society as a mechanism for advocacy within government were all highly constructive. 

Overall this project has identified several critical determinants that underpin a productive partnership approach to supporting implementation of FCTC Article 5.3 [11]. An engaged and responsive Ministry of Health, that appreciates that TII is present and undermines public health, was fundamental to the success of the project. Furthermore, developing legislation can benefit from access to clear and unambiguous guidelines and principles. Civil society involvement was similarly essential in the process of strengthening legislation for Article 5.3. Specifically, the role of civil society in undertaking the activities that raise public awareness and elicit wider public sector support raises expectations on the government to respond appropriately (in strengthening loopholes that have allowed TII, for example). Effective civil society involvement, as witnessed in the Solomon Islands, balances public expectations with the challenge of government to respond appropriately to clear public examples of TII. 

Two limitations to the study are worthwhile noting. In respect to the selection of PNG, Solomon Islands and Vanuatu, these three countries identified tobacco industry interference in-country and were willing to progress to the intervention phase of the project. Other countries indicated a willingness to participate, but did not opt to identify TII as a problem in country. By selecting countries from those that responded to the survey and that met the criteria for the project (known presence of TII, willingness to participate) this indicates that we were likely to be working with countries that had higher level support for the initiative and an ‘openness’ about the level of tobacco industry involvement in public policy. We conclude that other PICs may not yet be at the stage whereby there is political acceptance of the TII as a matter of public policy interest or may not have been directly affected by the tobacco industry. Secondly, the team members were generally known within the PICs for tobacco control support and have strong relationships in country. This may be another positive dimension of the initiative that may not be readily replicated. 

Public media plays a crucial role in exposing TII in a format that attracts both public and political attention and elicits an immediate response. The news media is a fundamental and cost effective tool for tobacco control advocacy—at this stage it remains the mainstream newspapers and radio, in the near future it will also include digital media, including social networks [17]. 

## 5. Conclusions

Progressing tobacco control in most countries requires substantial political commitment alongside sheer determination from civil society to maintain pressure on government to respond to TII [1,8,17]. However, as in many democratic jurisdictions, there were opportunities and leverage points presented from which to advance tobacco control policy in the Pacific Islands. From our analysis, there are two pathways for action, the background (relationship building, meeting with key officials, drafting of regulations, for example) and “the front of house” or the street where tobacco is sold and impacts are felt. The media plays a key role in tobacco control advocacy for change—timely and accurate reporting of key events, debates and controversy are all catalysts for public engagement on these issues [18]. 

However, even with all the above in place, it remains the prerogative of the country to commit to action, in this case, the adoption and implementation of the draft policies and legislation. On-going monitoring of the industry action is vital and requires a commitment across the wider public service sector in addition to health. Training public service officials to recognize and respond to TII will need to be embedded in induction and code(s) of conduct in order to ensure that there are no soft areas for TII to flourish. Again, leadership from an agency, ideally the Department of Health, is essential to drive the implementation and enforcement of legislation supporting FCTC Article 5.3. Finally, partnership based on trust and clear commitment to long-term support is a vital ingredient for progressing tobacco control in the Pacific.
